# Exploring reading profiles of rural school students

**DOI:** 10.1007/s11881-022-00276-y

**Published:** 2023-01-11

**Authors:** Johny Daniel, Amy Barth

**Affiliations:** 1 School of Education, Durham University, Leazes Rd, Durham, DH1 1TA UK; 2 William Jewell College, Liberty, MO USA

**Keywords:** Cognition, Latent profile, Reading disabilities, Reading profile, Rural

## Abstract

This study investigates the reading profiles of rural Grade 5 and 6 students (*N* = 262), a sample with a high proportion of English language learners. We administered a battery of reading and cognitive assessments to classify students’ reading profiles and evaluate if performance on cognitive measures predicted membership in particular profiles. Data were analyzed using latent profile analysis. Latent profile analysis showed four distinct reading profiles in our sample: students with severe reading disabilities (< 2%), students at high risk of reading disability (14%), students at some-risk of reading disability (46%), and students who are typical readers (38%). Lower performance on cognitive measures was associated with group membership in the severe reading profile group compared to the group of students at some-risk of reading failure. In contrast, higher performance on cognitive measures was associated with group membership in the typical reader group compared to students at some-risk of reading failure. In keeping with the findings from past studies documenting reader profiles, we found heterogeneity in the reading profiles of rural upper-elementary grade students. We discuss the need for multicomponent interventions that target all areas of reading with some flexibility in the dosage of each reading component dependent on the reader profiles established prior to intervention.

Compelling evidence links reading comprehension with improved educational outcomes, access to and appropriate use of health care resources, and greater short- and long-term employment opportunities (Aro et al., 2019; Berkman et al, 2011; Cortiella & Horowitz, 2014). A large body of evidence also suggests that reading comprehension represents a factor that mitigates intractable health disparities among marginalized and minoritized subgroups of children and adults in the USA (Paasche-Orlow & Wolf, 2007). Despite the well-intentioned efforts of reading researchers to improve reading outcomes for at-risk student populations such as students with reading difficulties and English language learners’ (ELs), results of reading interventions for students in the upper elementary and secondary grades largely indicate small to moderate effects (Hall et al., 2017; Scammacca et al., 2016). These findings suggest that although current interventions lead to practical and meaningful improvements in reading skills, they do not close the large performance gap that struggling readers must overcome to adequately comprehend grade-level texts (Cortiella & Horowitz, 2014; Faggella-Luby & Deshler, 2008).

To better understand students’ modest response to reading interventions, several researchers have conducted extant data analyses using intervention data. The analyses have demonstrated that pretest reading skills across different reading-related domains significantly influence response to treatment (e.g., Clemens et al., 2019; Daniel et al., 2021). For instance, Daniel et al. (2021) reported that in a sample of students with reading difficulties, individuals who started the intervention with lower word reading scores relative to peers demonstrated lower levels of growth on reading comprehension measures at the end of a year-long multicomponent reading intervention. This finding is consistent across multiple studies that report lower response to reading interventions for struggling readers who begin intervention with comparatively lower reading comprehension (Wanzek et al., 2016), word reading (Daniel et al., 2021), reading fluency (Clemens et al., 2019), and listening comprehension (Vaughn et al., 2019) skills. In short, these extant data analyses demonstrate that pre-intervention status is highly predictive of post-intervention response. Thus, reading interventions might be more effective at closing the performance gap if the instruction was more closely aligned to the specific strengths and weaknesses struggling readers present (i.e., reader profiles) at the initiation of treatment (see Connor et al., 2007, 2011).

## Need for research on reading profiles of rural students

A further concern is the general absence of research conducted with students attending rural school districts. That is, while several past studies have explored the profiles of students at-risk of reading failure, almost all analyses have used samples of students receiving educational services in urban or suburban settings (e.g., Capin et al., 2021).

This gap in the literature is noteworthy because approximately one in six of the total population of American students receives educational services in rural settings (McHenry-Sorber 2019). Past reports on reading proficiency in elementary and middle schools have shown that rural students’ reading performance is close to 1/3rd of a standard deviation lower when compared to their suburban peers (Graham & Teague, 2011). Furthermore, rural students do not close this achievement gap with their non-rural peers in later grades. For instance, in Grades 3 to 8, students from non-rural schools continue to outperform their peers in rural schools on reading-related tasks (Johnson et al., 2018). In the past, researchers have attributed rural students’ lower attainment to less credentialed teachers compared to non-rural schools (McHenry-Sorber, 2019), longer commutes that can lead to fatigue (Arnold et al., 2005), and lack of academic resources (Roscigno & Crowley, 2001). Other factors that can influence student academic performance in rural schools are the growing number of ELs and a greater percentage of students from economically disadvantaged and minority backgrounds compared to a decade earlier (Johnson et al., 2018).

Given that students in rural areas are a changing demographic and represent a significant proportion of the student population, it is important to understand their reading profiles to better inform research and practice in rural schools. The need for this paper arises from a scarcity of research literature involving rural schools; thus, we aim to better understand the reading profiles of a unique population of students in a rural school comprising a growing majority of ELs. To guide this work, we first present a theoretical framework. Next, we discuss important reading and cognitive skills to be considered prior to the initiation of treatment for ELs who read below grade-level benchmarks. Finally, we review the extant literature on reader profile analyses and how this might guide future interventions for ELs.

### Theoretical framework: components associated with reading comprehension

Theories guiding this study include the simple view of reading (SVR; Gough & Tumner, 1986) and the verbal efficiency theory (Perfetti, 1985). The SVR (Gough & Tunmer, 1986) emphasizes the importance of foundational component skills, such as word reading and linguistic comprehension, for comprehension of written texts. Both of these skills are multidimensional in nature and encompass clusters of subskills. For instance, successful word reading requires students to establish phonological awareness, build awareness of the alphabetic principle, and develop knowledge of phoneme-grapheme correspondences. Alternately, developing linguistic comprehension involves building background knowledge, developing vocabulary knowledge, cultivating awareness of language structures, and enhancing verbal reasoning skills. Failure to develop one or more of these subskills can be detrimental to proficiency in reading comprehension and can hinder independent learning from text (e.g., Daniel et al., 2021; Wanzek et al., 2016). Below, these reading component skills, which can be dissociated to determine whether students present weaknesses in word reading, linguistic comprehension, or both are reviewed.

### Word reading

It is well-established that word reading ability plays an influential role in comprehending text. In a meta-analysis, García and Cain, (2014) reported a strong correlation between word-reading ability and performance on reading comprehension measures (*r* = .74) for readers across age groups and reading levels. The association remained strong when data were disaggregated for poor word readers across different age groups (*r* = .64). Additionally, findings from longitudinal and cross-sectional studies provide evidence that word-reading ability in early-elementary grades is highly predictive of students’ reading comprehension outcomes in later grades (e.g., Shankweiler et al., 1999; Tighe et al., 2015). For instance, Tighe and colleagues (2015) reported that word-reading ability explained a significant proportion of variance in reading comprehension scores for all ability readers in third, seventh, and tenth grades. However, the proportion of variance explained was greater in third grade (56%) compared to seventh (34%) and tenth (24%) grades. These results indicate that word reading continues to play an important role in students’ ability to comprehend text and can be the leading cause of reading comprehension difficulties among students in the upper-elementary grade-levels.

Past studies that assessed the validity of the SVR generally reported that word reading plays a relatively important role in early-elementary grades, with linguistic comprehension making a larger contribution to reading comprehension in later grades (e.g., Tilstra et al., 2009). However, there are exceptions to this trend. For instance, students who are below-average readers continue to demonstrate word-reading deficits that account for variance in their reading comprehension in upper elementary and later grades (Cirino et al., 2012). For ELs, studies generally report word-reading abilities similar to non-EL peers (e.g., Lesaux et al., 2007; Mancilla-Martinez & Lesaux, 2011). In a longitudinal study of reading development, Lesaux et al. (2007) reported that although Spanish-speaking ELs performed slightly below their monolingual peers on some kindergarten academic measures, there were no significant differences between the two groups on word-reading measures in Grade 4. Similarly, in another longitudinal study, Mancilla-Martinez and Lesaux (2011) reported that growth rates, of 4- to 11-year-old English and Spanish-speaking students, on word-reading measures were similar to national norms. The similarity in growth on word-reading measures between ELs and non-ELs is generally attributed to cross-language transfers and overlap between phonology and orthography of English and ELs’ first language (Verhoeven, 2017). Thus, there is compelling evidence that word-reading ability develops similarly in Spanish-speaking ELs and their non-EL peers.

### Linguistic comprehension

The construct of linguistic comprehension encompasses multiple components such as vocabulary knowledge (both word and world) and listening comprehension. Several studies have demonstrated that linguistic comprehension, in addition to word reading, accounts for a significant proportion of the variance in reading comprehension (e.g., Language and Reading Research Consortium & Chiu, 2018; Lonigan et al., 2018). Results from past studies have also demonstrated that poor performance on listening-comprehension measures parallels difficulties with reading comprehension (Barnes et al., 1996; Keenan et al., 2006). Similarly, vocabulary knowledge has also been shown to account for the largest proportion of variance in reading comprehension (Ahmed et al., 2016; Cromley & Azevado, 2007). There is strong evidence that vocabulary knowledge predicts reading comprehension in elementary (Simmons & Kameenui, 1998), middle (Beck et al., 1982), and high school (Cunningham & Stanovich, 1997) grades. Thus, both vocabulary knowledge and listening comprehension are interconnected with readers’ background knowledge, and reading comprehension can be significantly impaired when the meaning of words in a text is unknown to the reader. It is noteworthy that while word reading explains a greater proportion of variance in early-elementary grades, linguistic comprehension has been shown to exert a greater influence on reading comprehension in upper elementary and later grade-levels (e.g., Kershaw & Schatschneider, 2012; Tilstra et al., 2009).

Literature on reading comprehension difficulties for ELs generally demonstrates greater deficits in areas of linguistic comprehension such as vocabulary knowledge compared to non-EL peers. In a recent study, Cho and colleagues (2019) utilized the SVR framework to investigate the mechanisms of reading comprehension for ELs and non-ELs. Using a sample of 440 below-average fourth-grade readers, authors reported that word reading was a major source of reading comprehension difficulties for non-ELs. In contrast, linguistic comprehension skills were identified as a major source of reading comprehension difficulties for ELs with significantly lower factor scores on vocabulary and oral comprehension measures compared to non-EL peers (Cho et al., 2019). Similar findings have also been reported for second-language learners in transparent orthographies (e.g., Verhoeven, 2000). Thus, research on second-language learners’ reading skills demonstrates that while word-reading ability develops at a similar pace compared to their monolingual peers, ELs’ development in linguistic comprehension skills such as vocabulary and oral comprehension lags behind their non-EL peers.

### Cognitive components

Process models and theories of reading comprehension, such as the verbal efficiency theory (Perfetti, 1985), help to explain reading and reading-related skills by which readers may be differentiated from one another (i.e., individual and developmental differences). Similar to the SVR (Gough & Tunmer, 1986), process models and theories emphasize the importance of word-reading skills and linguistic comprehension to reading comprehension. Of importance to this study, process models and theories also recognize that cognitive processes, such as working memory and non-verbal reasoning, play an important role in the reading and understanding written text (Peng et al., 2022). For example, the verbal efficiency theory (Perfetti, 1985) posits that when word reading is slow and labored, greater cognitive resources are allocated to decoding the text, leaving fewer cognitive resources available for comprehending the text. Furthermore, once the text has been decoded, students must “reason” with the text forming both text-to-text connections and text-to-knowledge connections.

#### Working memory

Studies that have assessed working memory in students have generally reported that students’ performance on working-memory tasks is associated with their word-reading and reading comprehension skills (Peng et al., 2018; Swanson et al., 2006). Limitations in working memory are considered to create a bottleneck that limits the reader’s capacity to retain recently processed knowledge to make connections to recent inputs. For instance, in the lexical-retrieval process, readers need to code text, store coded information, and accurately retrieve information from memory to make reliable connections between speech sounds and written text. During the lexical-retrieval process, it is theorized that readers visually recognize a sequence of letters as formulating a word and, thereafter, retrieve its phonological and semantic information from memory. Past researchers have utilized different methods to understand the lexical-retrieval process by either measuring students’ working memory (requiring students to store and process/manipulate the information) or measuring their short-term working memory (involving passive storage of information). An example of working memory is presenting students with a list of words and having them repeat the words in reverse order. In contrast, short-term working-memory tasks may involve students repeating the words in the order they were presented. Poor performance on both memory tasks has been associated with poor performance on reading measures (Swanson et al., 2006).

However, it is important to note that interventions focusing on improving students’ cognitive abilities, such as working memory, have not reported improved performance on academic outcomes. For instance, the results of a meta-analysis (Kearns & Fuchs, 2013) measuring the impact of cognitive-focused interventions on academic performance reported that there is not enough empirical data to support the use of cognitive-focused instruction for low-achieving students.

#### Non-verbal reasoning

Vital to the process of comprehension is readers’ ability to make connections between the information presented and their own prior knowledge relating to the content. The process of making such connections is referred to as the inferential process or inference making. To accurately infer, readers monitor their understanding of the content, relate the information presented to the material presented earlier in the text or their own knowledge base, and establish semantic associations supported by the text to generate accurate inferences. In other words, inference making requires readers to not only extract and construct meaning from the content being read but also to make connections within texts or between the text and their background knowledge (Barth et al., 2021; Kendou et al., 2008; Silva & Cain, 2015). Similarly, non-verbal reasoning tasks that require students to tap into their own knowledge to accurately respond to pictorial-inference tasks have also been associated with reading comprehension. Researchers have reported that non-verbal reasoning tasks significantly predict reading comprehension in typically developing elementary (Asbell et al., 2010), middle, and high school students (Tighe & Schatschneider, 2013). Researchers have also reported significant differences on non-verbal reasoning tasks between typical and struggling readers (Nation et al., 2002) supporting the notion that reasoning skills are vital to accurately infer from text and improve reading comprehension.

In this study, we focus on understanding the reading profiles of rural students with a large proportion of ELs. We create reading profiles utilizing students’ reading comprehension, word reading, vocabulary, and listening comprehension proficiency scores. Moreover, we test to ascertain whether cognitive measures such as working memory and non-verbal reasoning predict group membership.

### Past research on the profile of students with reading difficulties

Over the last two decades, researchers have used several statistical methods to better understand the various profiles of students struggling to comprehend grade-level texts (e.g., Buly & Valencia, 2002; Capin et al., 2021; Cirino et al., 2012; Clemens et al., 2019; Leach et al., 2003). One of the most consistent findings of these studies has been that students with reading difficulties comprise a heterogenous population with deficits in different areas of reading. While some students exhibit phonological deficits manifested in the form of labored and error-prone reading of the written language, others demonstrate deficits in extracting and constructing meaning from text, leading to poor comprehension of the content. Still, others may demonstrate deficits in both word reading and comprehension of grade-level texts.

Several studies have reported that students with reading difficulties in upper elementary and later grades display below-average skills in comprehension, word reading, reading fluency, or vocabulary knowledge, with deficits in comprehension and an additional reading domain being the most common (e.g., Cirino et al., 2012; Leach et al., 2003). Buly and Valencia (2002) analyzed the reading-skill profiles of 108 fourth graders identified as below-basic readers based on state-administered reading assessment scores. Using cluster analysis to create similar pairs of cases to identify reader profiles, authors reported that approximately 44% had deficits in reading comprehension and word reading/fluency. Additionally, 82% performed poorly on measures of decoding and/or reading fluency, while 58% performed poorly on measures of reading comprehension and vocabulary. Thus, a little over half of the sample demonstrated deficits in comprehension and vocabulary, while much of the sample exhibited deficits in word reading only. More recently, Capin et al. (2021) investigated the reading profiles of Grade 4 students with reading difficulties; all students in the sample scored < 85 on a standardized reading comprehension measure. The authors (Capin et al., 2021) reported that most of the participants comprising the at-risk reader sample (91%) scored approximately one standard deviation below the mean on measures of word reading and listening comprehension. A smaller proportion of the sample demonstrated either severe deficits in word reading (5%) or listening comprehension (4%).

Researchers have also explored reading-skill profiles of students with reading difficulties at the middle and high-school levels and reported reader profiles similar to those of third and fourth graders with reading difficulties. For instance, Cirino et al. (2012) used confirmatory factor analysis to study the reading profiles of 846 students with reading difficulties in Grades 6–8; participants were identified as students with reading difficulties when they failed to meet state reading comprehension proficiency-test benchmarks and scored below the 25th percentile on at least one standardized-reading measure. Reading-skill profiles of students with reading difficulties showed difficulties in decoding (47%), reading fluency (45%), and reading comprehension (84%). Additionally, 78% of the sample demonstrated difficulties in both fluency and comprehension.

Similar reading profiles of ELs have also been reported in the literature. For instance, in a sample of 87 Spanish-speaking students from upper-elementary grades, Lesaux et al. (2010) reported that participants demonstrated average word-reading skills but below-average oral-language skills. In their models, they illustrated that oral-language skills had a greater influence on ELs’ reading comprehension compared to word-reading skills (Lesaux et al., 2010). Similarly, in a longitudinal analysis of 150 elementary-grade ELs and non-ELs, Kieffer and Vukovic (2012) reported that among a subset of the sample with poor reading comprehension, 80% performed poorly on vocabulary and oral-language comprehension measures. While these studies provide evidence that linguistic comprehension accounts for a larger source of variability in reading comprehension for ELs, these findings are not consistent. In another longitudinal study of elementary-grade ELs, authors reported that, in Grade 5, word reading accounted for a larger proportion of variance in reading comprehension compared to vocabulary knowledge (Mancilla-Martinez & Lesaux, 2010). In the Capin et al. (2021) study, the authors were able to disaggregate the results for ELs with reading difficulties in their profile analysis. Their results demonstrated that while ELs were more likely to be in the listening-comprehension difficulties profile, they still accounted for a considerable proportion of the severe word-reading difficulties and the moderate reading/language difficulties profiles.

Together, these studies provide information on the heterogeneity of reading-skill profiles among students with reading difficulties. However, several studies comprising this body of literature used samples from either urban or suburban school districts to guide research and develop reading interventions for at-risk readers. Another challenge in interpreting the results of many past reading-profile studies concerns the use of a single reading-related measure to dichotomize the sample of students who were at-risk and not at-risk of reading failure. Past research does not empirically support using individual measures of reading to identify students with reading disabilities as single measures are imperfect indicators of underlying constructs (e.g., Fletcher et al., 2019; Miciak et al., 2015). A pertinent implication of identifying students with a single reading measure is the possibility of inaccurate inferences concerning profiles of struggling readers. Using different reading assessments across different studies can lead to different sets of students being identified with reading difficulties resulting from the manner in which tests operationalize the construct of reading (see, Miciak et al., 2015).

### Study purpose

The primary aim of this study is to understand the reading profiles of rural upper-elementary grade students with a high proportion of ELs. Results from this study can inform assessment, direct evaluation, and guide intervention development for at-risk students in rural areas, especially ELs. We also aim to overcome past methodological challenges involving the use of a particular measure to identify students who are at-risk or not at-risk of reading failure. To overcome this issue, we use the entire sample of students on a continuum of reading abilities to better represent the current diverse rural-student population.

### Hypotheses and research questions

No previous study has examined the reading profiles of rural Grade 5 and 6 students. We aimed to use an exploratory statistical method to investigate the various reading profiles of students in one rural school. Given that past studies have reported a range of reading profiles in their samples, we did not have any prior hypotheses on the number of reading profiles we would uncover in our sample. We did, however, expect to find students with generally low scores on vocabulary and listening comprehension due to the large proportion of ELs in our sample. Additionally, based on past studies’ findings (e.g., Capin et al., 2021), we hypothesized that students in the lower reading-performance profiles will also demonstrate lower performances on cognitive measures.

We investigate the following research questions: What are the reading profiles of upper-elementary grade students in a rural school district with a high proportion of ELs? Does performance on cognitive measures predict group membership?

## Method

### School

The school from which this study’s sample was derived is situated in a rural geographic location. The location of the school meets the definition of the term *rural* as any location that is not in an urban area (US Census Bureau). The county the school is located in is also categorized as a rural area (code 10) according to the rural–urban commuting area codes. The upper-elementary school this sample was derived from has a total enrollment of 789 students with 51% Hispanic, 17% Asian, 15% White, 6% Black, and other races (11%). Approximately, 64% of the student population identify as English language learners, and 75% qualify for free and reduced meals.

### Student participants

Data for this study were taken from pretests administered during a larger randomized controlled trial that included students in Grades 5 and 6 (Barth et al., 2021). Data were collected over two academic years from one school in a rural midwestern school district. In the first year, data were collected for Grade 5 and 6 students not at-risk of reading failure (considered typical readers). In the second year, data were collected for Grades 5 and 6 students considered at-risk of reading failure. Students’ reading-risk levels were determined using their district-administered FastBridge Adaptive Reading (aReading) scores (Christ, 2015). According to the FastBridge aReading benchmarks, students are considered at some-risk of reading failure when their composite scores are less than 513 and 517 in Grades 5 and 6, respectively.

### Measures

Trained undergraduate research assistants administered the measures except the Fastbridge aReading measure (Christ, 2015). The school administered the measure and provided the scores to the research team. To ensure fidelity of implementation, all undergraduate research assistants were required to administer all assessments with 100% accuracy to the primary investigator prior to beginning assessment with study participants. The primary investigator and project coordinator were also present in the school to answer questions during administration and to provide ongoing support as needed to ensure successful delivery of the group and individual assessment battery. All assessments were rescored twice by two different research assistants to ensure accuracy in scoring. To ensure fidelity of assessment for the Fastbridge aReading measure, which was administered by classroom teachers, the school district’s reading coach delivered training sessions to all teachers prior to administration and provided ongoing support during assessment delivery.

### Reading-related measures

#### FastBridge aReading

(Christ, 2015). FastBridge Adaptive Reading (aReading) is a computer-administered assessment. The aReading test measures students’ proficiency in various reading-related domains such as concepts of print, vocabulary, and comprehension of literary and informational text. The test requires students to answer questions related to the main idea and structure of the text. It also requires students to make inferences by integrating knowledge and ideas presented in the text. Students answer approximately 30 questions and receive a composite score based on their performance. The content, construct, and predictive validity of aReading were assessed by comparing results to other reading measures such as the Gates-MacGinitie reading comprehension test (MacGinitie et al., 2000) (median *r* = 0.78) and Measure of Academic Progress (MAP; Northwest Evaluation Association, 2016) (median *r* = 0.77). The reported alternate form reliability is 0.95, the internal consistency is 0.95, and the test–retest reliability ranges from 0.71 to 0.86.

#### Woodcock–Johnson Oral Comprehension

(WJ; Woodcock et al., 2001). The WJ oral comprehension subtest requires students to listen to orally presented sentences and short passages. Students then complete a sentence or short passage by orally providing the missing word that makes sense in the context. For example, given the verbal prompt, “A fish ______,” a student might say “swims” to accurately complete the sentence. The reported median test reliability for this subtest is 0.85.

#### Test of Word Reading Efficiency

(TOWRE-2; Torgesen et al., 2012). The TOWRE-2 sight word efficiency (SWE) subtest is a standardized, individually administered, timed test that requires students to read a list of printed words in 45 s. The test measures an individual’s ability to decode real words fluently. The phonemic decoding efficiency (PDE) subtest is also an individually administered timed test that gives students 45 s to read a list of pseudo words (e.g., ip, sline). The PDE subtest measures students’ ability to phonetically decode words fluently. The test–retest reliability is 0.90 for a sample of third- and fifth-graders, while alternative-form reliability exceeds 0.90.

#### Kaufman Brief Intelligence Test-2 Verbal Reasoning

(KBIT; Kaufman & Kaufman, 2004). The KBIT verbal reasoning measures students’ receptive and expressive vocabulary calculated using students’ scores on two subtests (i.e., verbal knowledge and riddles). In the verbal knowledge subtest, students are presented with an oral question and required to choose from one of six illustrations that best answers the question. In the riddles’ subtest, students are orally presented with a riddle and required to point to a picture or say a word that answers the riddle (e.g., point to something crunchy that elephants eat). Both subtests are discontinued when students provide four consecutive incorrect answers. Raw scores from both subtests are used to calculate a verbal reasoning standard score. The internal consistency for the test ranges from 0.90 to 0.93, and the test–retest reliability ranges from 0.85 to 0.88 for the age range of the sample in this study. The concurrent validity of the KBIT-2 verbal reasoning test and the Wechsler Abbreviated Scale of Intelligence’s vocabulary subtest (WASI; Wechsler, 1999) is 0.84 (Kaufman & Kaufman, 2004).

### Cognitive measures

#### Kaufman Brief Intelligence Test-2 Non-Verbal Reasoning

(KBIT-2; Kaufman & Kaufman, 2004). The KBIT-2 non-verbal reasoning subtest measures examinees’ fluid-reasoning and visual-processing abilities. Students are presented with a visual stimulus and have to decide which of the five pictures best match the stimulus picture. The number of pictures students choose from goes up to six as the test progresses. The matrices subtest is discontinued when a ceiling is reached (i.e., students provide four consecutive incorrect answers). Raw scores from the matrices’ subtests are used to calculate a non-verbal reasoning standard score. The internal consistency for this test ranges from 0.81 to 0.87, and the test–retest reliability ranges from 0.69 to 0.76 for the age range of our study sample. The concurrent validity of the KBIT-2 non-verbal reasoning test and the WASI matrix reasoning subtest (Wechsler, 1999) is 0.81 (Kaufman & Kaufman, 2004).

#### Woodcock–Johnson III Tests of Cognitive Abilities

(WJ; Schrank et al., 2001). We administered the WJ numbers reversed and the WJ memory for words subtest from the Test of Cognitive Abilities test battery. The WJ numbers reversed and the WJ memory for words have a median test reliability of 0.87 and 0.80, respectively. Both subtests are auditory tests that require the tester to read out a list of numbers or words and elicit oral responses from the test takers. In the numbers reversed subtest, the tester reads out a list of numbers (e.g., 3–8-6), and the test taker must reverse the sequence and repeat the numbers (e.g., 6–8-3). This subtest requires students to mentally manipulate numbers, which tests their cognitive flexibility and working memory. In the memory for words subtest, the tester reads out a list of unrelated words (e.g., mother, chair) and expects the test taker to repeat those words in the order they were presented. This subtest measures individuals’ short-term memory and attention.

### Data analysis

Latent profile analysis (LPA) was employed using covariance matrices of individuals to uncover latent groups of students’ reading profiles (Bauer & Curran, 2004). LPA allows researchers to investigate the different reader profiles using continuous indicators and analyze the predicted value of average scores on various continuous variables for members of individual profile groups. LPA analysis maximizes homogeneity within each profile as well as heterogeneity between subgroups. Importantly, these individual-profile subgroups are latent and not observed because membership is determined by investigating patterns of means and associations between indicator variables. In other words, the goal of LPA is to uncover latent subgroups of profiles (*k*) of individuals (*i*) who have similar patterns of responses or scores on indicator measures (*j*) (Ferguson et al., 2020; Sterba, 2013).

Similar to structural equation modeling (SEM), there are multiple steps involved in LPA analysis. The first step is to analyze a series of iterative models to determine the number of profiles to retain. We used the TOWRE SWE and PDE (word reading), FastBridge aReading (reading comprehension), WJ Oral Comprehension (language comprehension), and KBIT-Verbal Knowledge (vocabulary) to determine and index the underlying reading profiles of rural students in upper-elementary grades. We used standard scores for all measures except the FastBridge aReading. The FastBridge aReading data were available in an extended scale format, and using the measure in the reported format could confound findings because of the different *at-risk of reading* cutoff points for students in Grades 5 and 6. To overcome this issue, we first created a subset of our data for Grades 5 and 6 students and then converted the FastBridge aReading scores into z-scores, separately for each grade-level, prior to our analysis.

Next, model retention decisions, to determine the number of rural upper-elementary grade reader profiles, were done by examining various indices. As recommended in the literature, we used log likelihood value, Bayesian information criterion (BIC), sample-adjusted Bayesian information criterion (SABIC), and Akaike’s information criterion (AIC) (Ferguson et al., 2020). Across these four fit indices (i.e., log likelihood, BIC, SABIC, AIC), lower values indicate better fit.

We also used entropy as a measure of profile classification certainty with higher values indicating a better fit of participant profile for a given dataset. Some researchers have identified values of 0.80 or greater as a benchmark that indicates minimal uncertainty in the model (Tein et al., 2013). Finally, we also evaluated the model using the Bootstrap Likelihood Ratio Test (BLRT) and Lo–Mendell–Rubin Adjusted Likelihood Ratio Test (LMRT). Both the BLRT and LMRT are utilized to measure the differences between k-1 and k class models. Additionally, a *p*-value allows to test the difference between the two models with *p*-values < 0.05 indicating *k* class models as a significantly better fit compared to the *k*-1 class model. Most importantly, we relied on reading theories and prior work in this area to evaluate the reasonableness of the models.

Following the LPA analysis, and the determination of the optimal number of profiles to retain, we conducted the analysis to explore the differences between latent groups on covariate measures. It is important to note that covariate analysis has no impact on the creation of the profiles especially because this analysis is conducted after the optimal number of profiles has been determined (Marsh et al., 2009). To conduct the covariate analysis, latent classes were regressed on cognitive measures such as WJ working memory of words, WJ working memory of numbers, and KBIT non-verbal reasoning. We used a three-step process referred to as the Bolck–Croon–Hagenaars approach (BCH; Bolck et al., 2004). In the BCH approach, the first step is to determine the optimal number of latent profiles represented in the data without including the covariates. Next, students’ individual class probabilities are utilized to indicate their probability of membership into each latent reading profile. The third step is to conduct a multinomial logistic regression analysis to evaluate the association between cognitive predictors (i.e., working memory, non-verbal reasoning) and the likelihood of dummy-coded profile membership.

One of the key assumptions of LPA is local independence. MPlus automatically imposes local independence across latent profiles by constraining indicators to be uncorrelated for each latent class. Given this default mode, if the model fits well, then it can be inferred that the assumption was met. We used the maximum likelihood estimation with robust standard errors estimator (MLR) for the analyses. All analyses were conducted using Mplus 8.4 (Muthen & Muthen, 2017). We ran the LPA and covariate analyses using the Mplus syntax presented by Ferguson and colleagues (2020). Finally, all students in our sample were tested across measures at pretest, and there were no missing data.

## Results

Table 1 presents demographic data for the sample. Table 2 presents correlation data between reading and cognitive measures. Additionally, Table 2 presents the mean scores for each variable for the study sample.

**Table 1:** Demographic table

Demographics	*n*	Proportion
Grade		
Five	155	.59
Six	107	.41
Sex		
Female	142	.46
Male	120	.54
English language learner		
Yes	152	.58
No	110	.42
Ethnicity		
Asian	49	.19
Black	15	.06
Hispanic/Latino	135	.52
White	43	.16
Other	20	.07
Home language		
English	81	.31
Not English	178	.68
Not reported	3	.01

**Table 2:** Correlation and descriptive statistics for measures

Variables (constructs)	1	2	3	4	5	6	7	8
1. WJ oral comprehension (LC)								
2. FastBridge aReading (RC)	.**65**							
3. TOWRE sight word efficiency (WR)	**.41**	**.65**						
4. TOWRE phonemic decoding efficiency (WR)	**.32**	**.57**	**.79**					
5. WJ working memory – words (WM)	**.23**	**.30**	**.30**	**.32**				
6. WJ working memory – numbers (WM)	**.28**	**.44**	**.49**	**.51**	**.45**			
7. KBIT non-verbal reasoning (IA)	**.42**	**.52**	**.33**	**.26**	.12	**.32**		
8. KBIT verbal reasoning (Vocab)	**.73**	**.76**	**.51**	**.40**	**.24**	**.33**	**.52**	
*N*	262							
Mean	95.71	0.00	99.22	99.13	97.74	99.64	101.74	90.37
*SD*	16.91	0.99	15.80	15.74	19.35	17.87	16.05	16.44

Note: *LC*, listening comprehension; *RC*, reading comprehension; *WR*, word reading; *WM*, working memory; *IA*, intellectual ability; *Vocab*, vocabulary; *WJ*, Woodcock Johnson; *TOWRE*, Test of Word Reading Efficiency; *KBIT*, Kaufman Brief Intelligence Test

^*^Bolded correlation coefficients are significant at *p* < .01

### LPA model

**Fig. 1:**
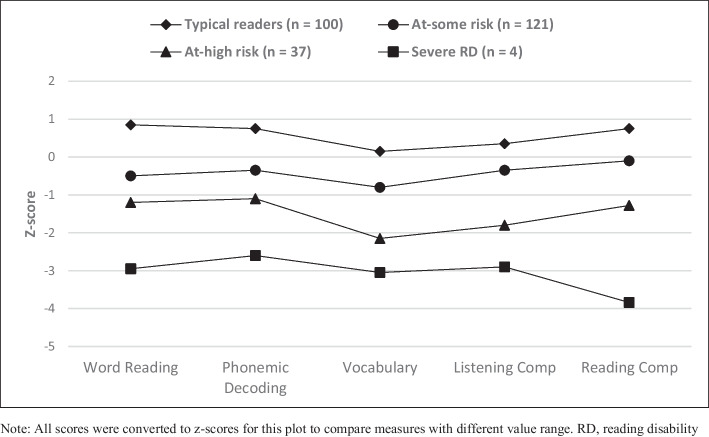
Line plot of average scores on reading measures for the four latent profiles

We selected a four-profile model solution because it was the only model with a significant LMRT when comparing *k* and *k*-1 models (see Table 3). Additionally, the four-profile solution also demonstrated the highest entropy (0.869). Table 4 and Fig. 1 present the estimated means for each reading-related measure for each of the four reader profiles. Results suggest that within our sample of Grade 5 and 6 rural-school students, there appears to be a small sample (*n* = 4; 1.5% of the sample) of students who have the most severe reading disabilities and perform approximately three standard deviations below the mean on all reading-related measures. The second profile (*n* = 37; 14% of the sample) includes students who scored approximately one standard deviation below the mean on word-reading measures such as TOWRE SWE (*M* = 82.73) and PDE (*M* = 84.08) and the reading comprehension measure (*M* =  − 1.28). On listening comprehension (*M* = 73.34) and vocabulary knowledge (*M* = 68.11), the second profile-cluster scored approximately two standard deviations below the mean. The third profile (*n* = 121; 46.5% of the sample) encompassed approximately half the sample of students in our study. This profile includes students who performed just below the mean on word-reading measures such as TOWRE SWE (*M* = 93.58) and PDE (*M* = 94.99), listening comprehension (*M* = 95.71), and reading comprehension (*M* =  − 0.10). However, this cluster of students seems to be at some-risk of reading failure due to their lower vocabulary mean estimate (*M* = 88.51). Finally, the fourth profile (*n* = 100; 38% of the sample) includes students who performed above average on all reading measures and can be considered typically developing readers.

**Table 3:** Latent profile analysis model fit summary

Model	Log likelihood	AIC	BIC	SABIC	Entropy	Class count	LMRT *p*-value	BLRT *p*-value
1	− 4775	9571	9607	9575	-	-	-	-
2	− 4587	9206	9264	9213	.803	91, 171	.093	< .001
3	− 4496	9037	9115	9046	.857	21, 127, 114	.469	< .001
**4**	− **4457**	**8970**	**9070**	**8982**	**.869**	**4, 37, 121, 100**	**.040**	** < .001**
5	− 4416	8901	9022	8914	.846	112, 5, 36, 58, 51	.179	< .001

*AIC*, Akaike’s information criterion; *BIC*, Bayesian information criterion; *SABIC*, sample-adjusted BIC; *LMRT*, Lo-Mendell Ruben test; *BLRT*, Bootstrap likelihood ratio test

Note: The BLRT and LMR test compare the current model to a model with *k* − 1 profiles

**Table 4:** Four-profile model results

Variable	Profile 1 Severe reading disability (*n* = 4)	Profile 2 At high risk of reading disability (*n* = 37)	Profile 3 At some risk of reading disability (*n* = 121)	Profile 4 Typical readers (*n* = 100)
Word reading				
- TOWRE SW	56.77 (2.04)	82.73 (1.86)	93.58 (2.01)	113.84 (1.42)
- TOWRE PD	61.39 (2.20)	84.08 (2.86)	94.99 (1.84)	111.21(1.16)
Vocabulary				
- KBIT – VK	54.06 (5.10)	68.11 (2.65)	88.51 (1.33)	102.24 (2.34)
Listening comprehension				
- WJ	57.36 (5.46)	73.34 (3.08)	95.71 (1.39)	105.45 (1.96)
Reading comprehension				
- FB	− 3.84 (0.74)	− 1.28 (0.16)	− 0.10 (0.06)	0.75 (0.10)

*FB*, FastBridge aReading; *WJ*, Woodcock–Johnson Oral Comprehension; *TOWRE*, Test of Word Reading Efficiency; *SW*, Sight Word Efficiency; *PD*, phonemic decoding efficiency; *KBIT-VK*, Kaufman Brief Intelligence Test Verbal Knowledge

Note: FB extended scale score converted to z-score by grade-level

### Cognitive predictors of profile membership

As shown in Table 5, based on the analysis, some covariates demonstrated a significant difference between profile 3 and the other three profiles. Results suggest that WJ working memory of numbers did not demonstrate any significant differences across the four reader profiles. In contrast, students with lower WJ working memory of words and lower KBIT non-verbal reasoning were more likely to be in the severe reading-disability profile (i.e., profile 1; *n* = 4) compared with students at some-risk of reading failure (i.e., profile 3). The reverse is true when comparing students at some-risk of reading failure (i.e., profile 3) to typical readers (i.e., profile 4); that is, higher scores on WJ working memory of words and higher KBIT non-verbal reasoning scores were more likely to be in the typical-reader profile compared to the at some-risk of reading failure profile. Finally, there were no differences on any cognitive measures between students at high risk of reading disability (i.e., profile 2) compared to students at some-risk of reading disability (i.e., profile 3).

**Table 5:** Cognitive covariate analyses results for the four-profile model

		Profile 3 (*n* = 121) vs		
Cognitive predictors		Profile 1 (*n* = 4)	Profile 2 (*n* = 37)	Profile 4 (*n* = 100)
WJ working memory words	Estimate (SE)	− 0.65** (0.15)	− 0.50 (0.35)	0.17** (0.04)
	Odds ratio (SE)	0.52** (0.16)	0.60 (0.21)	1.18** (0.04)
	Effect size	− 0.38	− 0.11	0.28
WJ working memory numbers	Estimate (SE)	− 0.04 (0.08)	− 0.08 (0.06)	0.01 (0.01)
	Odds ratio (SE)	0.96 (0.07)	0.92 (0.06)	1.01 (0.01)
	Effect size	− 0.04	− 0.10	0.07
KBIT non-verbal reasoning	Estimate (SE)	− 0.15* (0.05)	− 0.03 (0.04)	0.06** (0.02)
	Odds ratio (SE)	0.85* (0.06)	0.96 (0.04)	1.06** (0.01)
	Effect size	− 0.27	− 0.06	0.20

Note: *WJ*, Woodcock Johnson; *KBIT*, Kaufman Brief Intelligence Test

^*^p < .05; **p < .01

## Discussion

Using the SVR (Gough & Tunmer, 1986) and verbal efficiency theory (Perfetti, 1985) as a framework, the primary aim of this study was to understand the reading profiles of rural upper-elementary grade students with a high proportion of ELs. We also aimed to address past methodological limitations surrounding the use of single indicator models to identify students who are at-risk or not at-risk of reading failure and student samples that exclude diverse rural populations. To overcome both methodological challenges, this study utilizes multiple indicators, the full continuum of reading abilities, and was inclusive of the current diversity represented in rural US school districts. We examined the following research questions: What are the reading profiles of upper-elementary grade students in a rural school district with a high proportion of ELs? Does performance on cognitive measures predict group membership? Both questions are discussed below.

### Reading profiles of rural upper-elementary grade students

In general, the results of this study suggest that rural upper-elementary grade students largely fall into four distinct groups, with each group presenting a unique reader profile. We found that a small proportion of the sample (i.e., 1.5%) had a severe reading disability (i.e., profile 1). Profile 1 students performed three standard deviations below the mean on measures of word reading, vocabulary, listening comprehension, and reading comprehension. This flat and pervasively low profile is indicative of severe reading disabilities, possibly comorbid with oral-language weaknesses (Kornilov & Grigorenko, 2018). It is highly likely that students in profile 1 have limited grade-level academic vocabulary, restricted world knowledge, and constrained word knowledge thus capping their ability to make the types of inferences needed to understand the grade-level text. Also, because profile 1 students have limited word-reading skills, they may likely struggle to decode familiar words both accurately and quickly. Similarly, their poor phonemic-decoding skills suggest that they may struggle to decode unfamiliar words in isolation and in text. As a result, profile 1 students may struggle to read connected text accurately, quickly, and with appropriate expression. Students presenting profile 1 may benefit from intensive reading intervention, spanning multiple years, targeting the component skills of word reading (accuracy and fluency) and linguistic comprehension to meet grade-level reading benchmarks (Roberts et al., 2015).

Results suggest that approximately 14% of the sample was at “high-risk” for reading failure (i.e., profile 2). Profile 2 students performed approximately one standard deviation below the mean on measures of word reading and passage comprehension and two standard deviations below the mean on measures of listening comprehension and vocabulary. Profile 2 students are not as severely impaired as profile 1 students; however, the gap separating this group from their typically developing peers (i.e., profile 4) is relatively large. Their significant linguistic-comprehension (i.e., vocabulary and listening comprehension) difficulties suggest that they struggle with the acquisition, retention, and use of academic language while reading and listening. Academic language is an essential component of content area disciplines in the upper-elementary grades. From understanding a class lecture, to engaging in large-, small-, or peer-group discussions, to reading for understanding, academic language is a central feature of instruction, linguistic comprehension, and independent learning (Proctor et al., 2019). Because the language of upper-elementary grade instructional texts is increasingly complex, profile 2 students may benefit from an intervention that targets their language-related reading difficulties while also bolstering their lower-than-average word-reading skills.

Almost half the sample (i.e., 46.5%) demonstrated “some-risk” of reading failure (i.e., profile 3). Profile 3 students performed slightly lower than typically developing students in profile 4, scoring approximately ¼ standard deviation below the mean on measures of word reading, listening comprehension, and reading comprehension. Of concern, profile 3 students scored approximately ¾ standard deviation below the mean on vocabulary. It is not surprising to see that ELs as well as monolingual English-speaking students with reading difficulties struggle with vocabulary and academic language of the classroom since it represents “a constellation of high-utility language skills that correspond to linguistic features that are prevalent in academic discourse across school content areas and infrequent in colloquial conversation” (Uccelli et al., 2015, p. 338). For this reason, profile 3 students may benefit from intervention that emphasizes vocabulary and comprehension as well as broader dimensions of language.

Finally, results suggest that approximately 38% of students in our sample were typically developing (i.e., profile 4). Profile 4 students performed above the mean on all measures. Interestingly, we were unable to detect a smaller subgroup of advanced readers that has been historically reported in the National Assessment of Educational Progress (NAEP) reading reports. Results may indicate generally lower-proficiency peaks among students who are educated in rural school districts. The lack of an advanced-reader profile may also be due to the higher proportion of ELs in this sample, who have historically demonstrated lower levels of reading proficiency relative to their typically developing monolingual peers (National Center for Education Statistics, 2020). It is also the case that the absence of an advanced-reader profile is unique to this sample and replication is required to improve the generalizability of findings.

### Predictive nature of cognitive abilities

The second question addressed in this study was the extent to which performance on cognitive measures predicted group membership. While profile analyses yielded four distinct reading profiles, cognitive abilities significantly differentiated students in profile 1. Specifically, students with the most severe reading disabilities (profile 1) performed poorly on cognitive tasks such as working memory for words and inferential processing compared to students at some-risk of reading failure (profile 3). Cognitive abilities also significantly differentiated students in profile 3 from typically developing students in profile 4. Students in profile 3 performed slightly but significantly lower than students in profile 4 on working memory for words and inferential processing.

Our findings of struggling readers’ poor performance on cognitive measures raise important questions about how students’ performance on cognitive measures can guide research and practice. Past research on the effects of cognitive-focused interventions on academic outcomes for at-risk student populations has been inconclusive (Kearns & Fuchs, 2013). There is little support to warrant implementing working memory interventions in classrooms to positively impact students’ reading-related outcomes. Elliott and Grigorenko, (2015) recommend that classroom instructors be sensitized to students’ working-memory shortcomings and provide modifications/accommodations to students. One method that has shown some success is to explicitly teach metacognitive strategies to help students structure their reading-related tasks (e.g., García-Madruga et al., 2013). Thus, it may be more beneficial to focus intervention efforts on more malleable aspects such as explicitly teaching metacognitive strategies as part of reading instruction to overcome working memory shortcomings.

### Connections to theory and past research

Both the SVR (Gough & Tunmer, 1986) and verbal efficiency theory (Perfetti, 1985) provide useful heuristics for guiding research and practice. A theme that emerges from these two theoretical perspectives is that word-level knowledge and discourse-level knowledge have consequences for the meaning-making processes that support comprehension. Specifically, meaning-making is strongly influenced by knowledge components: knowledge about words (i.e., grammatical forms, spelling, and pronunciations) and their multiple meanings (Perfetti, 2007). Reading experience provides opportunities to integrate meaning extracted from information located within and between sentences with meaning retrieved from our general knowledge stored in long-term memory. These meaning-making and connecting processes operate during reading to create a mental representation of the situation described by the text (Kintsch, 1988). The resulting mental representation or situation model includes content from the text that is integrated with the reader’s specific topical knowledge to provide an evolving understanding of what has been read.

Individual differences in comprehension are not only the result of the size of one’s store of general knowledge. Another source is the stability of the knowledge that the reader has about word forms and meanings and their capacity to efficiently retrieve and integrate this knowledge to construct coherent representations of the text (Barth et al., 2021; Perfetti, 2007). Less stable knowledge or the lack of knowledge will impose constraints on one’s ability to generate inferences while reading, maintain both local and global textual coherence, and independently learn from the text (Barnes et al., 1996, 2015; Barth et al., 2021). Working memory, which is utilized to efficiently integrate information within and between sentences with general knowledge, also represents a reader characteristic that can impose constraints on comprehension (van den Broek et al., 2005).

Given the high proportion of ELs in this sample and three reader profiles suggesting deficits in verbal knowledge, what do we know? First, longitudinal studies examining the relationship between verbal knowledge and reading comprehension show that early word knowledge predicts later comprehension among elementary-age children (de Jong & van der Leij, 2002; Oakhill & Cain, 2012; Storch & Whitehurst, 2002). Among secondary-grade students, a cross-sectional study demonstrates the direct and substantial effects of word and world knowledge on reading comprehension (Ahmed et al., 2016). Second, in studies of struggling comprehenders with intact word-reading skills, difficulties with vocabulary extend beyond simply knowing the meaning of words. Even when these readers have learned the meaning of new words, they may continue to experience difficulties efficiently accessing its meaning to form inferences while reading (Cain et al., 2001). Third, research related to ELs consistently shows that vocabulary and linguistic comprehension are significant sources of difficulty (Cho et al., 2019). As a result, ELs require additional practice trials to learn new words, and once new vocabulary is mastered, they do not consistently use this knowledge to form inferences while reading to maintain coherence (Barth et al., 2021). Finally, according to Paris (2005), word and world knowledge represents an unconstrained skill. This means that our knowledge base starts to develop before we learn how to read words and continues to grow during as well as after learning how to read for understanding. However, many ELs develop English language proficiency while also learning how to read in English (Klinger et al., 2006). As such, their limited English word and world knowledge is often compounded by difficulties in efficient access to this knowledge in working memory thereby reducing their ability to both establish and maintain understanding while listening or reading (Perfetti, 2007).

### Implications for future research

There is considerable evidence that students who are at-risk of reading failure form a heterogenous population that have deficits in multiple reading-related domains (e.g., Capin et al., 2021). These findings seem to remain consistent with urban (e.g., Cirino et al., 2012; Leach et al., 2003), suburban (e.g., Clemens et al., 2019), and now rural samples. The heterogeneity in reading profiles also seems to be consistent regardless of the language status of the reader (i.e., EL or non-EL) (O’Connor et al., 2018). Thus, interventions that target students with reading deficits must align instruction to meet the needs of students’ pre-intervention reading profiles and be multicomponent in nature. These suggestions align with recent findings of moderation analyses of experimental studies that have tested the effects of multicomponent reading interventions on reading-related outcomes for at-risk student populations. Extant data analyses of empirical data have consistently shown that intervention effects vary dependent on pretest performance on key reading-related measures such as word reading, listening comprehension, reading fluency, vocabulary, and reading comprehension (e.g., Clemens et al., 2019; Daniel et al., 2021; Vaughn et al., 2019). Furthermore, there is also some evidence from intervention research that demonstrates, when intervention is aligned with individual students’ needs there are greater effects of the intervention on students’ reading outcomes (Connor et al., 2007, 2011). For instance, Connor and colleagues (2011) demonstrated that students who were assigned to treatment groups that individualized instruction to their reading needs performed significantly better at posttest than their peers who received a standardized reading intervention.

Given the diverse student reading profiles we uncovered in one school, it is important to develop and test the efficacy of multicomponent reading interventions (e.g., Vaughn et al., 2021) that are customizable to suit the needs of this heterogenous population depending on pre-intervention reading profiles. That is, there is a need to develop multicomponent interventions that can be tailored to meet the needs of different reader profiles. While standardized interventions may help teachers implement the program with fidelity, adaptable interventions that meet the evolving needs of students may prove to be effective (Elliott & Grigorenko, 2015).

Our findings also align with past research demonstrating that students in upper-elementary grades continue to struggle with word-reading proficiency (Cirino et al., 2012), and there is a great need to develop word-reading interventions for this population. Additionally, more research is needed in addressing the needs of students with the most severe reading disabilities. The drastically low scores of this small sample of students should challenge reading interventionists to explore and design reading programs and curricula that are embedded in theoretical frameworks and innovative in design and application.

Finally, descriptive statistics (see Table 4) illustrates that all four reader profiles on average scored lowest on the vocabulary measure compared to other reading-related measures. Given the high proportions of ELs in our sample and the historically low performance of ELs on vocabulary measures (Cho et al., 2019; Kieffer & Vukovic, 2012), it is vital for classroom instruction to focus on providing regular explicit instruction in academic language that includes vocabulary and morphology instruction. Instruction should focus both on knowledge acquisition as well as how to use new knowledge productively while reading. This two-pronged approach to instruction will help struggling readers to close the gap between “knowing” and strategically integrating knowledge to form inferences while reading, using the knowledge to monitor comprehension, and drawing on the knowledge to fix breaks in understanding. Thus, future research should continue to develop a critical mass of language-focused curricula that promotes deeper engagement with academic vocabulary to improve at-risk students’ lexical representations.

### Limitations

A limitation of the current study is that the sample was derived from one school in a rural school district. Although our findings are mostly consistent with past profile studies, replication studies with other linguistically diverse rural samples are needed to generalize these findings. Furthermore, data for our analyses were collected from one cohort of typical readers and one cohort of at-risk readers across 2 years and combined for our analyses. However, descriptive statistics for the combined cohorts showed normally distributed data with skewness and kurtosis in the acceptable range for all cognitive and reading-related measures.

Additionally, we were unable to administer and did not have access to students’ scores on multiple measures of each construct to create latent variables for our analyses. We also did not have access to measures of certain important reading-related constructs such as phonological awareness, spelling, and reading fluency to provide a more comprehensive view of each reading profile. Furthermore, although a majority of our sample was ELs (58%), we did not have enough power to run latent profile analysis for this subsample of students. Finally, to answer our primary research question, we used an exploratory statistical technique (i.e., LPA), and future research should cross-validate these rural upper-elementary grade reader profiles with larger multi-site samples.

## Conclusions

In summary, like past studies, we found heterogeneity in the profiles of rural upper-elementary grade readers. Profile 1 (severely disabled) emphasized the need for multi-year, intensive interventions focused both on word reading and linguistic comprehension. Cognitive skills such as inferential processing and working memory for words also suggested limited capacity which is required for successful and efficient integration of information within and between sentences while reading and listening for understanding. Profile 2 (moderate-risk) and profile 3 (some-risk) highlight the need for improved general education instruction and intense interventions for students educated in the rural setting, particularly ELs. Fine-grained differentiation was observed with linguistic comprehension included in the latent profiling especially with the inclusion of vocabulary. Interestingly, the profiles missing are the classic dyslexic profile wherein students have well-developed linguistic-comprehension skills but are below-average on code-related skills and an advanced-proficient profile demonstrating superior mastery of basic reading and language skills associated with reading comprehension. Given the variation in students’ reading skills, it may be that standardized and homogenous reading programs may be less effective in meeting the needs of a heterogenous population of readers. Thus, we suggest designing and implementing multicomponent reading interventions that vary the dosage of reading components dependent on individual reader profile needs, vary the size of the instructional group, and the duration of the intervention.

## Data Availability

The data that support the findings of this study are available on request from one of the co-authors. The data are not publicly available due to privacy or ethical restrictions.
